# ITGAV and ITGA5 diversely regulate proliferation and adipogenic differentiation of human adipose derived stem cells

**DOI:** 10.1038/srep28889

**Published:** 2016-07-01

**Authors:** E. M. Morandi, R. Verstappen, M. E. Zwierzina, S. Geley, G. Pierer, C. Ploner

**Affiliations:** 1 Department of Plastic, Reconstructive and Aesthetic Surgery, Medical University of Innsbruck, Anichstrasse 35, 6020 Innsbruck, Austria; 2 Department of Anatomy, Histology and Embryology, Medical University of Innsbruck, Müllerstrasse 59, 6020 Innsbruck, Austria; 3 Division of Molecular Pathophysiology, Medical University of Innsbruck, Innrain 80, 6020 Innsbruck, Austria

## Abstract

The fate of human adipose tissue stem cells (ASCs) is largely determined by biochemical and mechanical cues from the extracellular matrix (ECM), which are sensed and transmitted by integrins. It is well known that specific ECM constituents influence ASC proliferation and differentiation. Nevertheless, knowledge on how individual integrins regulate distinct processes is still limited. We performed gene profiling of 18 alpha integrins in sorted ASCs and adipocytes, identifying downregulations of RGD-motif binding integrins integrin-alpha-V (ITGAV) and integrin-alpha-5 (ITGA5), upregulation of laminin binding and leukocyte-specific integrins and individual regulations of collagen and LDV-receptors in differentiated adipocytes *in-vivo*. Gene function analyses in *in-vitro* cultured ASCs unraveled differential functions of ITGA5 and ITGAV. Knockdown of ITGAV, but not ITGA5 reduced proliferation, caused p21^Cip1^ induction, repression of survivin and specific regulation of Hippo pathway mediator TAZ. Gene knockdown of both integrins promoted adipogenic differentiation, while transgenic expression impaired adipogenesis. Inhibition of ITGAV using cilengitide resulted in a similar phenotype, mimicking loss of pan-ITGAV expression using RNAi. Herein we show ASC specific integrin expression patterns and demonstrate distinct regulating roles of both integrins in human ASCs and adipocyte physiology suggesting a negative impact of RDG-motif signaling on adipogenic differentiation of ASCs via ITGA5 and ITGAV.

In regenerative medicine, the exertion of influence on cell viability and *in-vivo* differentiation is of great interest, as reconstructing complex soft tissue defects still remains a major clinical challenge. Tissue engineering techniques, extracellular matrix (ECM) scaffolds and the application of multipotent adipose derived stem cells (ASCs)^1^ are largely investigated attempts in preclinical and translational research. However, knowledge about possible external influence on ASC physiology as well as clinical experience in this field is still limited. Although ASCs and their developmental potential are well characterized, the molecular basis for *ex-vivo* expansion and application of these cells for the purpose of tissue engineering or specific clinical applications in regenerative medicine remains unclear. An upcoming body of literature describes multiple effects of the extracellular matrix (ECM) on MSC and ASC physiology, including proliferation and differentiation. The ECM impacts on these functions by specific molecular composition and mechanical properties^2^,^3^,^4^. Characterizing interactions between cells and the ECM is therefore crucial for *in-vitro* expansion and differentiation of MSC as well as ASC.

ASCs interact with the surrounding microenvironment mainly through integrins^5^, a protein family that comprises 18 α-subunits and 8 β-subunits in mammals^6^, which form at least 24 heterodimers of one α- and one β-subunit^7^. Upon binding to specific components of the ECM, integrins undergo a conformational change and form focal adhesions^7^. Associated intracellular protein complexes consequently control numerous cellular developmental processes by modulating transduction signaling cascades^8^,^9^ such as PI3K-PDK1-AKT or MEK-ERK pathways and impact on F-actin dynamics via the regulation of Rho-GTPase activity^8^. More recently, the Hippo pathway, an evolutionarily conserved pathway that controls tissue growth by the regulation of cell proliferation, differentiation and cell death has been connected to integrin-dependent adhesion^10^,^11^,^12^,^13^. Controlled by extracellular mechanical cues such as ECM rigidity or cell-cell contacts, the Hippo pathway mediates its signaling by modulating the expression and activity of the two major downstream effectors Yes-associated protein (YAP) and transcriptional co-activator with PDZ-binding motif (TAZ). Both proteins act as transcriptional co-factors controlling the expression of Hippo pathway target genes such as connective tissue growth factor (CTGF)^14^ or survivin^15^. All of the mentioned signaling pathways are involved in the regulation of proliferation, migration and differentiation and thus plainly able to influence cell destiny^16^,^17^ and tissue development. Therefore, a fundamental understanding of matrix-integrin interactions is important in order to elucidate basic ECM requirements of ASCs.

Previous studies have shown that cell proliferation of primary ASCs is favored by the presence of RGD-motif containing substrates such as fibronectin or vitronectin^18^. Signals from those ECM constituents are mainly recognized by integrin-alpha-5 (ITGA5) and integrin-alpha-V (ITGAV). While ITGAV serves as a subunit for integrin receptors binding RGD-motif containing substrates such as vitronectin, fibronectin and fibrinogen^19^, ITGA5 is mainly part of fibronectin- and osteopontin binding receptors^20^. Thus far, ITGAV has been shown to play an important role in the regulation of cancer growth and metastasis^21^. Especially the ITGAV/ITGB3 heterodimer has been connected with tumor neoangiogenesis via high levels of bFGF and tumor-necrosis factor α (TNFA) whereas the ITGAV/ITGB1 receptor is implicated in tumor cell proliferation via BCL2 and p53 activity^22^. However, the role of ITGAV as a fibronectin receptor and its impact on ASC cell physiology and adipogenic differentiation remains poorly defined.

To continue the research into the role of integrins in tissue remodeling, we herein analyzed the integrin expression profile of sorted primary ASCs and adipocytes *in-vivo*. We identified RGD-binding integrins as being consistently repressed during adipogenesis and outlined differential roles for ITGAV and ITGA5 in ASC proliferation and differentiation. We defined a molecular mechanism how these integrins diversely regulate ASC proliferation and showed that the specific phenotype can be induced by pharmacological inhibition of ITGAV signaling using cilengitide.

## Results

### Integrin expression patterns of differentiated adipocytes and ASC

**Figure 1:**
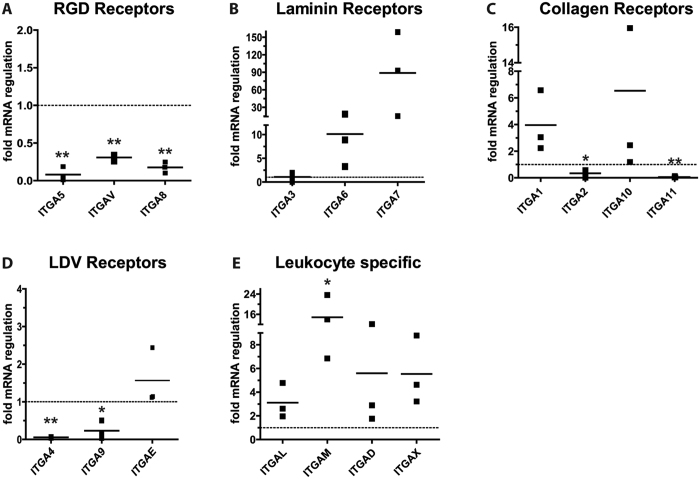
Integrin regulation in adipogenesis. *In-vivo* integrin expression was analyzed in sorted primary human ASCs (CD34^+^/CD90^+^/CD31^−^/CD45^−^) and adipocytes isolated from subcutaneous fat tissue of three donors employing quantitative RT-PCR. Values are depicted as mean fold change in gene expression (2^−ΔΔCT^) of adipocytes compared to ASCs (adipocyte/ASC ratio). Integrins were grouped according to their main binding motif: (**A**) RGD-motif binding specific integrins, (**B**) laminin recognizing integrins, (**C**) collagen specific integrins, (**D**) LDV-motif recognizing and (**E**) leukocyte specific integrins. Data were normalized to the geometric mean of the reference genes GUSB and YWHAZ. Asterisks indicate p-values < 0.001 (**) or <0.05 (*).

To determine integrin expression patterns, we isolated samples of primary adipocytes and ASCs (CD34^+^/CD90^+^/CD31^−^/CD45^−^) for quantitative RT-PCR analysis of 18 known alpha integrins. Multipotency of the isolated ASCs was confirmed by *in-vitro* trilineage differentiation (Supplementary Fig. 1). Comparison of integrin expression levels in ASCs and adipocytes revealed that RGD-motif recognizing integrins, ITGA5, ITGAV, ITGA8 and ITGA2b were strongly repressed in differentiated adipocytes (Fig. 1A). ITGA5 mRNA decreased more than 14-fold (mean fold regulation: 0.08 ± 0.09, p < 0.001), whereas ITGAV (mean fold regulation: 0.30 ± 0.05, p < 0.001) and ITGA8 (mean fold regulation: 0.17 ± 0.07, p < 0.001) expression levels decreased more moderately. The expression levels of ITGA2b mRNA were beyond the detection limit. In contrast to the downregulation of RGD-motif recognizing integrins, laminin receptors ITGA6 (mean fold regulation: 10.1 ± 7.6, p = 0.105) and ITGA7 (mean fold regulation: 88.8 ± 72.0, p = 0.102) were strongly upregulated in differentiated adipocytes (Fig. 1B). ITGA3 (mean fold regulation: 1.07 ± 0.9, p = 0.927), was not regulated during adipogenesis. Of the alpha-I-domain containing integrins that predominantly recognize collagen, ITGA1 (mean fold regulation: 3.9 ± 2.3, p = 0.0913) and ITGA10 (mean fold regulation: 6.5 ± 8.1, p = 0.308) were induced, while the expression of ITGA2 (mean fold regulation: 0.34 ± 0.29, p = 0.017) and ITGA11 (mean fold regulation: 0.06 ± 0.05, p < 0.001) was reduced in differentiated adipocytes (Fig. 1C). Integrins binding to an acidic motif termed “LDV”^23^ that is present in fibronectin but also in other ligands such as VCAM1 and MadCAM1, ITGA4 (mean fold regulation: 0.06 ± 0.01, p < 0.001) and ITGA9 (mean fold regulation: 0.23 ± 0.24, p = 0.005) were repressed in terminally differentiated adipocytes (Fig. 1D), while ITGAE was slightly induced upon differentiation (mean fold regulation: 1.6 ± 0.75, p = 0.2689). The last group of integrins, namely leukocyte specific integrins ITGAL (mean fold regulation: 3.1 ± 1.5, p = 0.067), ITGAM (mean fold regulation: 14.8 ± 8.4, p = 0.047), ITGAX (mean fold regulation: 5.5 ± 2.9, p = 0.053) and ITGAD (mean fold regulation: 5.6 ± 5.7, p = 0.236) were induced on mRNA level upon differentiation in all donors (Fig. 1E). However, compared to the cycle of threshold levels of reference genes, the overall mRNA levels of these integrins were very low, with CT-values above 30 in sorted ASCs. In summary, adipogenesis related regulation of integrins was dominated by the repression of RGD-recognizing integrins and collagen receptor ITGA11 as well as strong upregulation of the laminin receptors ITGA6 and ITGA7 and leukocyte specific receptor ITGAM.

### RGD-receptors ITGA5 and ITGAV are repressed during adipogenesis

Previous studies have demonstrated, that culture of MSC on fibronectin or RGD-peptide coated plates promotes proliferation but not differentiation^18^. We found that all RGD-motif binding integrins were repressed during adipogenesis *in-vivo*, and therefore focused on ITGAV and ITGA5 for functional analyses. Pharmacological inhibitors^24^,^25^ have been designed for both integrins and are currently being tested in clinical oncologic studies, which further prompted us to investigate their possible functional significance for tissue restoring applications.

**Figure 2:**
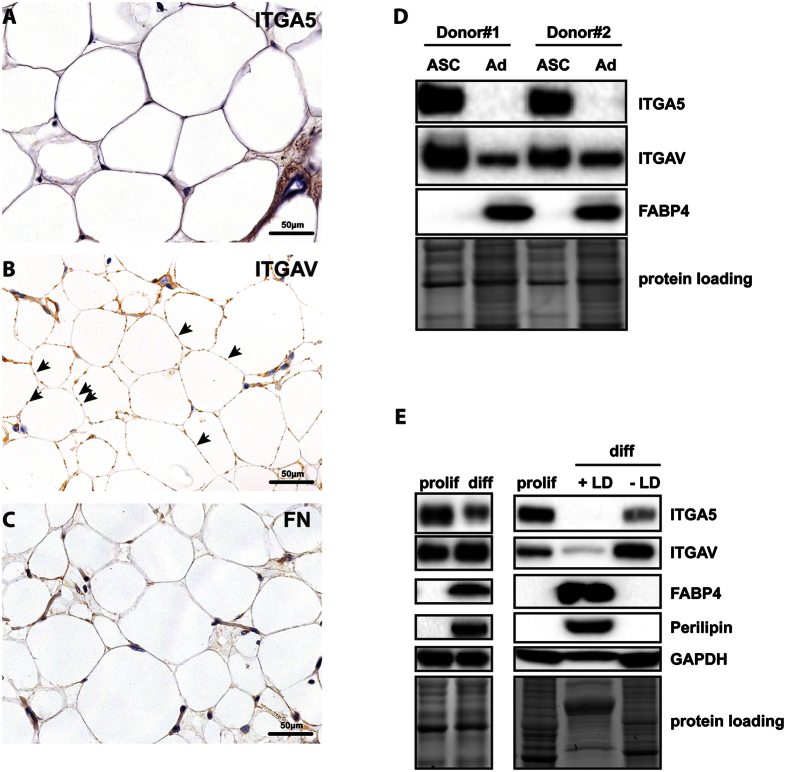
Expression of fibronectin receptors ITGAV and ITGA5 *in-vivo* and *in-vitro*. Immunohistochemistry analysis of paraffin fixed human subcutaneous fat tissue sections using specific antibodies against (**A**) ITGA5, (**B**) ITGAV and (**C**) fibronectin. Adipocytes can be morphologically identified as “signet ring cells” containing large fat vacuoles in the images. Protein expression was visualized using horseradish peroxidase (brown) and hematoxylin counterstaining (blue). Note the small ITGAV protein accumulations indicated by arrows at connecting adipocyte membranes (**B**). (**D**) Sorted ASCs and primary adipocytes of two representative donors (Donor#1 and Donor#2) were subjected to immunoblotting analyzing the expression of ITGA5, ITGAV and adipocyte specific marker FAPB4. (**E**) Cell lysates of *in-vitro* differentiated ASCs (day 14, left panel; proliferating cells are labeled with “prolif”, differentiated cells are labeled with “diff”) and the same cells separated according to their lipid droplet content (LD, right panel; lipid droplet containing cells are labeled with “+LD”, cells containing no lipid droplets are labeled with “−LD”) were subjected to immunoblotting detecting ITGAV, ITGA5, GAPDH and the adipocyte specific markers FABP4 and PLIN1. As loading controls of the depicted immunoblots cropped images of total protein stains acquired before blotting are shown. Full length gels are presented in Supplementary Fig. 4.

First, we confirmed mRNA expression data by immunohistochemistry and immunoblot analysis of sorted ASCs and primary adipocyte lysates. ITGA5 was strongly expressed in undifferentiated cells, but downregulated in mature adipocytes (Fig. 2A,D), which can be morphologically identified as “signet ring cells” in tissue sections and reveal a homogenous intracellular, peripheral expression pattern for adipocyte specific marker PLIN1 (Supplementary Fig. 3). ITGAV levels were similarly high in undifferentiated cells (Fig. 2D). However, expression was not completely abolished in mature adipocytes (Fig. 2B,D). Instead, residual protein accumulations at specific sites between adipocyte membranes were observed. The dotted pattern might indicate either development of cell-cell contacts to enhance the mechanical properties of the tissue or existence of RGD-motif containing ECM constituents surrounding adipocytes. Testing the second possibility, fibronectin staining showed increased levels in the ECM of undifferentiated cells but did not stain protein accumulations at adipocyte-adipocyte contact sites (Fig. 2C).

To investigate whether the observed regulations were also detectable in *in-vitro* differentiated cells, we subjected ASCs to adipogenesis and determined ITGA5 and ITGAV expression by immunoblotting. ITGA5 expression in *in-vitro* differentiated adipocytes decreased, corresponding to our *in-vivo* observations. Surprisingly, ITGAV expression was slightly induced upon *in-vitro* differentiation, which we suspected to be due to the amount of non-differentiated cells (Fig. 2E, left panel). To enrich differentiated cells, lipid droplet containing cells were selectively isolated by means of an OPTIPREP^TM^ based density centrifugation method^26^ and subjected to immunoblotting (Fig. 2E, right panel). Similarly to *in-vivo* differentiated adipocytes, both integrins were strongly downregulated in differentiated cells. In contrast, cells subjected to adipogenesis that did not develop sufficient numbers of lipid droplets also showed reduced ITGA5 levels, but intriguingly showed an upregulation of ITGAV, which might explain increased ITGAV levels in unseparated samples. In summary, the results supported our mRNA data showing a decline in expression of both integrins during adipogenesis and therefore suggest an essential function in adipogenic differentiation.

### Loss of ITGAV moderately induced cell death and reduced cell proliferation

**Figure 3:**
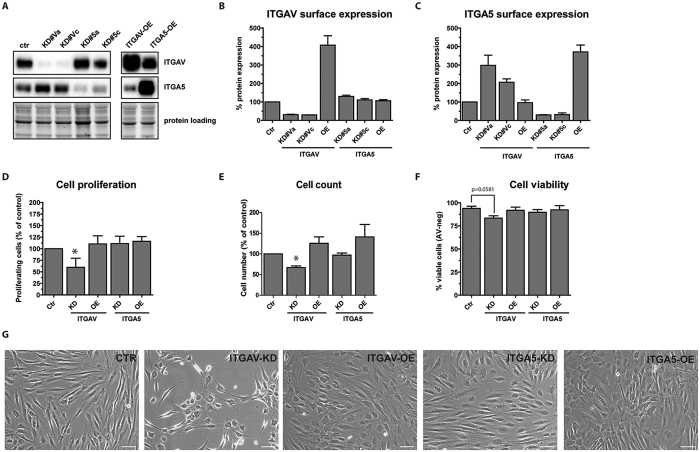
Effect of ITGAV and ITGA5 knockdown and overexpression on cell proliferation and viability. (**A**) Efficacy of two individual shRNAs designed to target ITGAV (labelled KD#Va for ITGAV-shRNA#a and KD#Vc for ITGAV-shRNA#c) and ITGA5 (labelled KD#5a for shRNA#a and KD#5c for ITGA5-shRNA#c) was determined by immunoblotting and flow cytometry analysis of ITGAV (**B**) and ITGA5 (**C**) expression in knockdown and overexpression cells five days after infection. (**D**) Proliferation of transduced ASCs was assessed by Prestoblue^®^ proliferation assay and automated cell counting (**E**) at day five after infection. (**F**) The same cells were analyzed for cell viability by flow cytometry of AnnexinV/PI-stained cells. (**G**) Representative phase microscopy pictures of transduced ASCs, five days after infection. Data represent the mean ± SD of five experiments targeting each integrin with two independent shRNA sequences. Presented data of KD-cells have been pooled (**D–F**). Asterisks indicate p-values < 0.05. As loading controls of the depicted immunoblots cropped images of total protein stains acquired before blotting are shown. Full length gels are presented in Supplementary Fig. 4.

To further investigate the importance of ITGA5 and ITGAV in ASC physiology, we designed two independent shRNAs for each integrin and cloned them into lentiviral GATEWAY^TM^ RNAi vectors^27^. For overexpression experiments, ITGA5 and ITGAV were expressed from corresponding lentiviral vectors (Fig. 3). In ASCs transduced with the RNAi vector, ITGA5 and ITGAV total protein levels were repressed to less than 10% of basal protein levels in control-shRNA (Ctr-shRNA) transduced cells (Fig. 3A). Analysis of ITGA5 and ITGAV cell surface levels by immunostaining and flow cytometry showed an average knockdown (KD) efficacy of 75–80% (Fig. 3B,C). Interestingly, we observed a compensatory upregulation of ITGA5 surface expression in ITGAV depleted cells. Vice versa, ITGAV was moderately upregulated in ITGA5-KD cells. As this regulation was not detected on mRNA level, increased synthesis, transport or protein stabilization of ITGA5 might occur in ASCs upon loss of ITGAV.

As the cell morphology of ITGAV-KD cells differed from that of ITGA5-KD or Ctr-shRNA infected cells (Fig. 3G), we further analyzed cell proliferation and viability. Automated cell counting (Fig. 3E) and mitochondrial activity as a marker of cell proliferation (Fig. 3D) showed a strong decrease in cell proliferation in ITGAV-KD cells at day five post infection. In contrast, ITGA5 depletion did not significantly reduce cell numbers or proliferation. However, at later time points loss of ITGA5 resulted in similarly low cell numbers suggesting a cell proliferation regulating role of ITGA5 in ASCs by continuous depletion (Supplementary Fig. 2). Analysis of cell viability by annexinV/PI staining in transduced ASCs (Fig. 3F) showed a moderate, but not significant increase in apoptotic cell numbers (mean: 9.5% ± 6.8, p = 0.19) in ITGAV-KD but not ITGA5-KD cells, indicating that some cells undergo cell death upon loss of ITGAV within the investigated time.

### Intracellular signaling pathways differentially regulated by ITGAV and ITGA5

**Figure 4:**
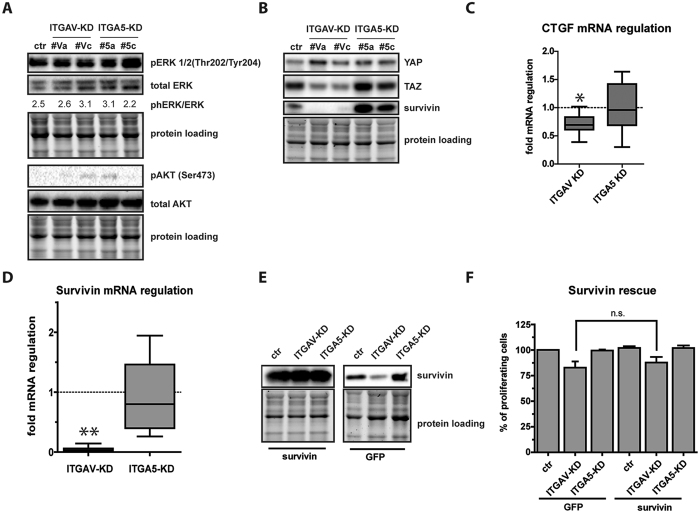
Intracellular pathway analysis links ITGAV signaling to the Hippo pathway. For the analysis of ITGAV- or ITGA5 controlled downstream pathways, phosphorylation of MAP-kinase ERK1/2 and serine/threonine kinase AKT (**A**) as well as expression of the Hippo-mediators YAP, TAZ and the TAZ-target gene Survivin (**B**) were analyzed in ITGAV- and ITGA5 depleted cells by immunoblotting using specific antibodies. (**C**) Quantitative RT-PCR analysis of the TAZ target genes CTGF and survivin (**D**) in ITGAV- and ITGA5 knockdown cells (fold mRNA regulation 2^−ΔΔCT^) compared to control infected cells. Data of KD-cells have been pooled. Asterisks indicate p-values < 0.001 (**) or <0.05 (*). (**E**) Survivin- or Ctrl-cDNA transduced ASCs were superinfected with ITGAV- or ITGA5-targeting shRNA and analyzed for transgene expression. Samples derived from the same experiment and were processed in parallel. Data show representative immunoblots at different exposure times (left panel 2 seconds, right panel 5 minutes). (**F**) Cell proliferation of transgenic survivin or GFP transduced cells superinfected with ITGA5- or ITGAV-targeting shRNA was assessed five days after infection (n.s. = not significant). Data represent the mean ± SD of 3 independent experiments. As loading controls of the depicted immunoblots cropped images of total protein stains acquired before blotting are shown.

Ligand binding to integrins activates many signaling pathways, including MAPK, AKT and Hippo pathways following SRC/FAK activation. One of the most cited pathways affected by integrin signaling is mediated by extracellular signal-regulated kinases 1/2 (ERK 1/2)^28^, which is controlled by MAP kinase-kinase (MAP2K) mediated phosphorylation at Thr202/Tyr204^29^. Analysis of phospho-ERK1/2 in proliferating ITGAV- and ITGA5-KD cells did not show integrin specific differences in ERK1/2 phosphorylation at Thr202/Tyr204, indicating that this signaling pathway was not responsible for the observed cell cycle effects (Fig. 4A). Next we analyzed S473 phosphorylation of AKT, as previous findings suggested a PI3-kinase dependent regulation of AKT upon integrin signaling at this phosphorylation site^30^. However, AKT phosphorylation was almost undetectable in transduced ASCs and no integrin-specific differences could be observed (Fig. 4A). Next, the expression of the two major downstream effectors of the Hippo signaling cascade, YAP and TAZ, was analyzed. Immunoblotting showed that loss of neither ITGAV nor ITGA5 exerted an influence on YAP levels (Fig. 4B), while TAZ levels were about 2-fold lower in ITGAV-KD cells than in ITGA5-KD or control infected cells. To evaluate whether this decrease affected the expression of known TAZ target genes we determined levels of CTGF and survivin in these cells by quantitative RT-PCR. As expected, CTGF levels showed a moderate decrease in ITGAV-KD cells. More impressive, survivin was strongly repressed in ITGAV but not in ITGA5-KD cells (Fig. 4B,D). The data suggested that ITGAV signaling is linked to the Hippo pathway by regulation of TAZ levels and repression of survivin, a cell cycle regulator and caspase inhibitor^31^, might be responsible for impaired proliferation in ITGAV-KD cells. Therefore, we performed rescue experiments by introducing transgenic survivin-cDNA or GFP-cDNA as control in ITGAV-KD cells and analyzed cell proliferation (Fig. 4E). Although survivin expression strongly overwhelmed levels in GFP-transduced cells, transgenic survivin was insufficient to rescue the proliferation phenotype induced by ITGAV-KD (Fig. 4F). This suggested that repression of survivin was not causative for the decrease in cell proliferation.

### Loss of ITGAV mediated upregulation of p21^Cip1^

**Figure 5:**
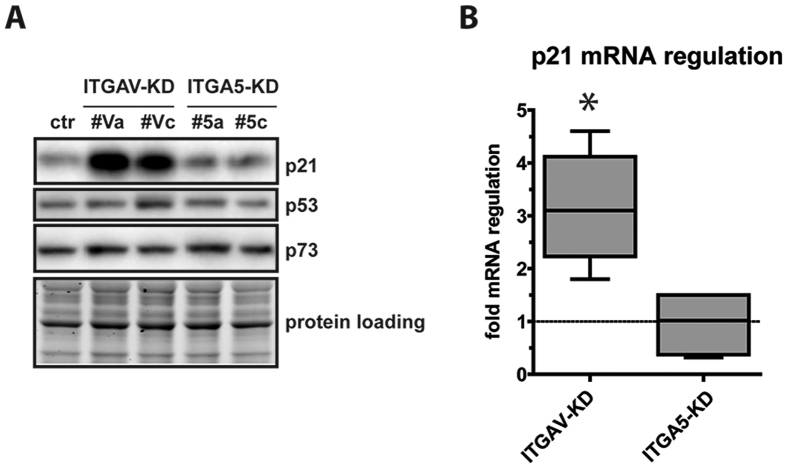
p21^Cip1^ is upregulated upon loss of ITGAV. (**A**) Representative immunoblotting of p21^Cip1^, p53 and p73 in ITGAV- and ITGA5-KD cells 5 days after infection. (**B**) Quantitative RT-PCR analysis of p21^Cip1^ mRNA in ITGAV- and ITGA5-KD cells expressed as fold change in gene expression. Data of KD-cells have been pooled. Shown data represent regulation values from four independent experiments, the asterisk indicates p < 0.01. As loading controls of the depicted immunoblots cropped images of total protein stains acquired before blotting are shown.

Since the so far investigated pathways did not sufficiently explain reduced cell proliferation upon loss of ITGAV, we investigated levels of cyclin-dependent kinase inhibitor 1A (p21^Cip1^), which has been shown to be stabilized upon disruption of filamentous actin (F-actin)^32^. Interestingly, we observed an up to 4-fold upregulation of p21^Cip1^ mRNA and protein levels in ITGAV-KD (p = 0.0072) but not in ITGA5-KD cells (Fig. 5A,B). As it is known, that the members of the tumor suppressor protein p53 family^33^ are regulators of p21^Cip1^, we determined levels of p53 and p73. As shown in Fig. 5A, neither p53 nor p73 levels increased in ITGAV-depleted cells, suggesting alternative induction of p21^Cip1^ in ITGAV- and ITGA5-KD cells.

### Loss of ITGAV promotes adipogenic differentiation of ASC

**Figure 6:**
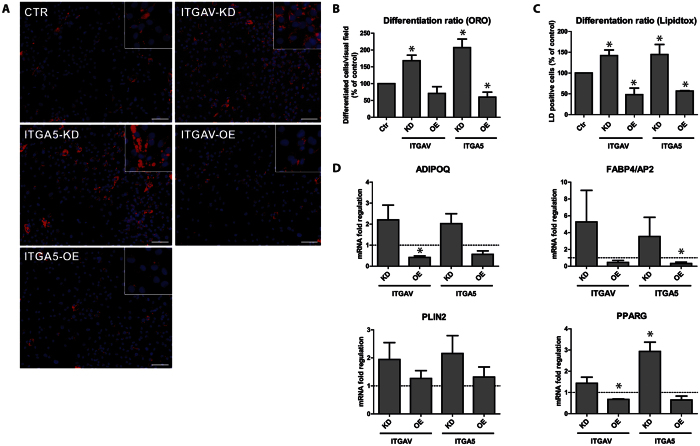
ITGAV and ITGA5 expression impairs adipogenesis. (**A**) Representative microscopic images of knockdown and overexpressing cells subjected to *in-vitro* adipogenic differentiation for 14 days. Lipid droplets and nuclei were visualized by Oil Red O (ORO, red) and Hoechst 33342 (blue) staining of paraformaldehyde fixed cells. (**B**) The number of differentiated cells was determined by counting of differentiated cells per visual field. A minimum of 5 pictures was analyzed per group in each independent experiment. (**C**) Cells from the same experiment were stained with HCS-LipidTOX^TM^-Green and differentiation was analyzed by flow cytometry, determining extent of green fluorescence. Shown data represent the mean ± SD of 5 independent experiments. Data of KD-cells have been pooled. (**D**) Quantitative RT-PCR analysis of adipocyte marker genes ADIPOQ, FABP4/AP2, PLIN2 and PPARG was performed at day 14 of differentation, shown data represent the mean ± SEM of 3 independent experiments. Presented data of KD-cells have been pooled. Asterisks indicate p-values < 0.05.

Previous studies have already shown that expression of ITGA5 counteracts adipogenic differentiation^34^. Moreover, pericellular fibronectin levels strongly decrease during adipogenesis, essentially connecting fibronectin signaling to the differentiation process^34^,^35^. Since ITGAV levels declined, but were not as strongly repressed as ITGA5 in adipogenesis, we functionally tested whether loss of ITGAV induced a similar phenotype in differentiating ASCs as loss of ITGA5. Vice versa, we performed gain of function experiments by constitutively expressing transgenic ITGAV- or ITGA5 in these cells to elucidate whether inhibition of differentiation is mediated by integrin expression. Transduced cells were subjected to adipogenesis and the formation of lipid droplets and expression of classical adipocyte marker genes were analyzed. Microscopy and flow cytometry analysis of LipidTOX Green stained lipid droplets revealed that loss of ITGA5 increased the numbers of lipid droplet containing cells to up to twofold. Similarly, depletion of ITGAV enhanced adipogenic differentiation as well, but the increase was more moderate. Vice versa, transgenic expression of ITGA5 or ITGAV reduced the numbers of well differentiated cells up to 60% (ITGAV-KD, p = 0.032) and 50% (ITGA5-KD, p = 0.030), respectively (Fig. 6B). In agreement, mRNA levels of adipocyte marker genes peroxisome proliferator-activated receptor γ (PPARG), lipid droplet associated protein 2 (PLIN2), adipocyte specific fatty acid binding protein (FABP4) and adiponectin (ADIPOQ) evidenced the phenotypic observations. PPARG showed significantly lower levels in ITGAV-OE cells than in control cells (p < 0.0001) and significantly higher levels in ITGA5-KD cells (p = 0.0117). Although not significant in all groups, levels of these marker genes in KD-cells clearly exceeded those in Ctr-shRNA transduced cells in all instances, corresponding well with the morphologic phenotype. Therefore, this might hint for enhanced activation of adipogenesis regulating signaling in these cells. In summary, the data demonstrate that loss of ITGAV and ITGA5 induced a similar but not necessarily the same differentiation phenotype, thus arguing for slightly distinct differentiation signaling mediated by these integrin subtypes.

### Pharmacological inhibition of ITGAV/B3 and ITGAV/B5 by cilengitide mimics loss of ITGAV in ASC

**Figure 7:**
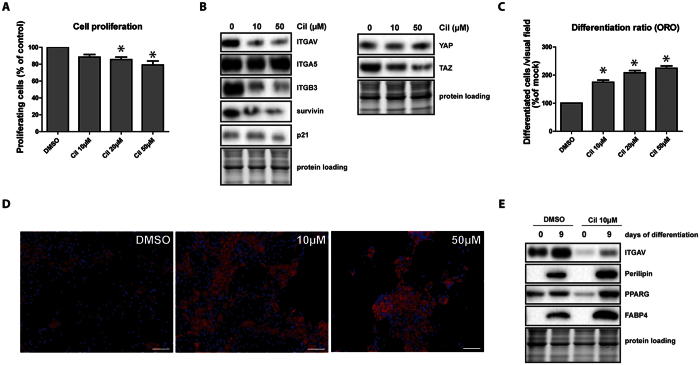
Pharmacological inhibition of ITGAV/B3 and ITGAV/B5 with cilengitide mimics loss of ITGAV. (**A**) Proliferation of cilengidite treated ASCs was assessed by Prestoblue^®^ proliferation assay 48 h after treatment. (**B**) Immunoblot analysis of ITGAV, ITGA5, ITGB3, survivin, p21^Cip1^, YAP and TAZ in ASCs treated with different concentrations of cilengitide for 48 h. (**C**) ASCs were subjected to adipogenic differentiation for 14 days and different concentrations of cilengitide were added to the medium. Extent of differentiation was visualized by Oil Red O staining (ORO) of paraformaldehyde fixed cells and assessed by counting differentiated cells per visual field. Asterisks indicate p-values < 0.05, a minimum of 5 pictures per group were analyzed in each experiment. Shown data represent the mean ± SD of 3 independent experiments. (**D**) Representative microscopic images of cilengitide or DMSO treated ASCs subjected to *in-vitro* differentiation for 14 days showing ORO stained lipid droplets (red) and Hoechst stained nuclei (blue), scale bar: 50 μm. (**E**) Cell lysates of day 14 differentiated ASC exposed to 10 μM cilengitide or control treatment (DMSO) were subjected to immunoblotting for expression analysis of the adipocyte specific markers FABP4 and PLIN1. As loading controls of the depicted immunoblots cropped images of total protein stains acquired before blotting are shown.

After assessing the importance of ITGAV and ITGA5 for the fate of mesenchymal progenitor cells isolated from human fat, we aimed to study the clinical feasibility of our observations. Therefore, we compared the effects of ITGAV-KD and the pharmacological inhibition of ITGAV/ITGB3 and ITGAV/ITGB5 complexes using ITGAV heterodimer inhibitor cilengitide, a molecule^36^ originally developed for the treatment of glioblastoma. Cilengitide was added to proliferating or differentiating ASCs each time the medium was changed. The effect on proliferating cells was similar to that observed in ITGAV-KD cells, showing decreased cell proliferation (Fig. 7A). ITGAV and ITGB3 protein levels decreased after administration of 10 μM cilengitide, corresponding to about 1/5 of a therapeutic dose^37^ in a normal weight patient (Fig. 7B). At this concentration, TAZ and survivin levels were repressed, correlating with our observations in ITGAV-depleted cells. Interestingly, neither ITGA5 counter-upregulation nor p21^Cip1^ accumulation was observed in cilengitide treated cells. In addition to the cell proliferation inhibiting effects, cilengitide promoted adipogenesis (Fig. 7C,D). Higher concentrations (20 μM, 50 μM) even enforced the phenotype, but also induced alterations in cell morphology and increased cell clustering indicating possible cell-toxic effects. At the molecular level, PPARG, PLIN1 and FABP4 were strongly induced in adipogenesis following cilengitide treatment, correlating well with the microscopic phenotype. In summary, pharmacological inhibition of ITGAV heterodimers phenotypically and molecularly corresponds to the loss of ITGAV and promotes adipocyte differentiation of ASCs.

## Discussion

The aim of the study was to identify essential regulators of matrix-cell interactions in ASCs that control cell fate and discriminate them from mature adipocytes. By systematically analyzing the expression of a wide panel of alpha integrins, which specifically recognize and bind ECM substrates, we elaborated expression profiles for sorted CD34^+^/CD90^+^/CD31^−^/CD45^−^ ASCs enriched from the SVF and primary adipocytes isolated from human subcutaneous tissue. Our data confirmed findings of previous *in-vitro* studies showing that integrins such as ITGA2, ITGA4^16^, ITGA5 and ITGA6^38^ are differentially expressed in ASCs and adipocytes. In addition we also unraveled new adipogenesis-specific integrin regulations. For instance, ITGA7 was markedly upregulated in differentiated adipocytes. Similar to ITGA6, ITGA7 specifically binds to laminin, a substrate that is an important ECM component of adipocytes containing large fat vacuoles^16^,^34^. Since antibody-mediated inhibition of ITGA6 does not alter the differentiation phenotype^34^, ITGA7 might be responsible for laminin-dependent signaling in differentiating preadipocytes. Furthermore we found that RGD-motif and LDV-recognizing integrins were consistently repressed during adipogenesis, whereas laminin and collagen receptors as well as the leukocyte specific integrins were upregulated in adipocytes. From these data it is tempting to conclude that the *in-vivo* niche of ASCs is rich in matrix substrates containing RGD-motifs like fibronectin or vitronectin and is poor in laminin subtypes, as ASCs indeed express RGD-binding integrin subtypes at high levels but hardly express laminin recognizing integrins. However, the complexity of ECM architecture and the availability of distinct substrate isoforms that additionally mediate mechanical cues might dampen this simplified view.

Among RGD receptors, ITGA8 mRNA expression was lowest in ASCs, but as ITGA5 and ITGAV were highly expressed in ASCs and strongly repressed upon differentiation, we focused on these integrins for functional analyses. ITGA5 has previously been shown to be repressed upon differentiation in 3T3-L1 preadipocytes^34^, whereas this effect could not be observed in *in-vitro* differentiated hMSC from the bone marrow^16^. To our knowledge, ITGAV has not yet been linked to adipogenic differentiation, but was shown to be important in the neural invasion of malignant tumors^39^, tissue fibrosis^40^ and neoangiogenesis^41^,^42^. Although mRNA levels of ITGAV were clearly lower in differentiated adipocytes, small amounts of ITGAV protein could be detected in patches between adipocytes *in-vivo*, suggesting an important role in maintaining tissue architecture and the three-dimensional ECM of adipose tissue.

Knockdown of ITGAV but not ITGA5 reduced of cell proliferation. It has recently been demonstrated that ITGAV is required for the maturation of focal adhesions, whereas ITGA5 controls early attachment and assembly of nascent adhesions. Our data might postulate a functional role of ITGAV in cell cycle^43^. ITGAV depletion was associated with a p53-independent induction of cell cycle regulator CDKN1A, the gene encoding p21^Cip1^ protein, suggesting that ITGAV expression might be required for proliferation following cellular adhesion. In contrast, although knockdown of ITGAV impaired adhesion, it only marginally increased apoptosis due to anoikis, suggesting functional redundancy to ITGA5 or other integrins.

In contrast to our findings in ASCs, proliferation inhibiting effects of ITGA5 have been described previously for a variety of cell types such as keratinocytes^44^ or muscle cells^45^. Although transgenic expression of ITGA5 increased proliferation in ASCs, the effect of RNAi mediated ITGA5 depletion on proliferation was not significant during the first six days after transduction. At later time-points proliferation levels approximated the level of proliferation in ITGAV-KD cells (Supplementary Fig. 2). These findings suggest that integrin signaling varies depending on the cell type. Under cell culture conditions used in this study the effect of transgenic ITGA5 expression might not be as significant as it might be for cells that are additionally cultured in the presence of exogenous RGD-containing substrates. On the other hand, this may be valid only for very early effects as adherent cells immediately begin to produce their own extracellular matrix upon attachment^46^.

To search for responsible intracellular signaling mechanisms for the phenotype we screened for activation of the ERK1/2 pathway as ERK1/2 signaling was previously shown to regulate actin polymerization^47^. In our experiments ERK phosphorylation at Thr202/Tyr204 did not depend on the differential expression of individual integrins, suggesting that ITGA5 and ITGAV signaling are not mediated by distinct activation of the MAPK-pathway - a finding that also holds true for the analysis of the AKT pathway. Contrarily, we found that the major Hippo pathway mediator TAZ but not YAP was significantly repressed in ITGAV-KD cells and TAZ repression went along with a reduction in survivin expression. Since Hippo signaling is involved in fundamental processes like proliferation arrest, contact inhibition, sensing of mechanical cues and cytoskeletal changes^14^, it is not surprising that this pathway is affected by ITGAV depletion. Although transgenic survivin did not rescue ITGAV-cells from reduced growth, the data strongly suggest – to our knowledge for the first time - a functional link between the Hippo pathway and integrin signaling. Whether the classical Hippo pathway cascade via LATs1/2 activation is involved or TAZ downregulation is due to another mechanism is the subject of current investigations.

The strongest effect of ITGA5/ITGAV knockdown was an increase in differentiation. Vice versa we observed adipogenesis inhibiting effects of transgenic ITGA5/ITGAV expression. In agreement with other authors^35^,^48^ who described a negative impact of fibronectin signaling on adipogenesis before, our experiments show significantly enhanced differentiation of ASCs upon loss of ITGA5 or ITGAV, mimicking the absence of fibronectin signaling. Vice versa, overexpression of both integrins strongly impaired adipogenesis. Although depletion of ITGAV decreased cell proliferation and slightly increased cell death at early time points after infection of proliferating cells, ITGAV-KD cells subjected to adipogenesis effectively underwent differentiation without any signs of cell death. In fact, cells plated to undergo differentiation have to be seeded at high density to induce a proliferation stop by cell contact inhibition. From this observation, we conclude that loss of ITGAV primarily affects proliferating ASCs and has no effect on the viability of arrested cells.

Based on the finding that ITGAV knockdown is able to enhance adipogenesis as described above, we tested whether ITGAV/ITGB3 and ITGAV/ITGB5 heterodimer inhibitor cilengitide is able to mimic this phenotype. As expected, pharmacological inhibition of ITGAV mimics total loss of ITGAV expression in both cell phenotype and intracellular signaling in a dose-dependent manner. Intriguingly, although all other downstream targets investigated showed regulations similar to that found in ITGAV-depleted cells, p21^Cip1^ levels were not increased following cilengitide treatment. Even longer exposure to the drug did not increase levels of this cell regulator, thus suggesting differential intracellular signaling. A possible explanation for this finding might be that shRNA-mediated ITGAV targeting affects the expression of all ITGAV heterodimers and is not restricted to ITGAV/ITGB3 and ITGAV/ITGB5, implying that upregulation of p21^Cip1^ in ITGAV-KD cells might result from the lack of ITGAV/ITGB1 and ITGAV/ITGB6 heterodimers.

In summary, we identified ASC specific integrins and functional analyses demonstrated that integrins expressed in primary ASCs and adipocytes contribute to cellular processes by influencing intracellular signaling pathways. Our data also strongly suggest a negative impact of RDG motif signaling on adipogenic differentiation of ASCs via ITGA5^34^ and - to our knowledge - for the first time via ITGAV.

## Methods

### Isolation and cell culture of human ASC

ASCs were isolated from subcutaneous abdominal fat tissue obtained from patients (mean age 36.2 ± 15.3 years; 88% female, 12% male) undergoing elective abdominoplasty. The study was approved by the Ethics Committee of the Medical University of Innsbruck (UN4368; EK 301/4.5), written informed consent was obtained from all donors and the methods were carried out in accordance with the approved guidelines. For ASC isolation, adipose tissue was washed with phosphate-buffered saline (PBS), minced into pieces and incubated with collagenase Type I (0.15% in PBS, Roche, Germany) for 1 h at 37 °C. After digestion, samples were centrifuged at 500 × g for 10 min. The upper phase containing primary *in-vivo* differentiated adipocytes^49^ was transferred into a new tube, washed with PBS and immediately subjected to RNA- and protein-isolation. Pelleted stromal vascular fraction (SVF) was treated with erythrocyte lysis buffer (0.5 M NH_4_Cl, 0.5 M KH_2_PO_4_, 100 mM EDTA, Roth, Germany) for 10 min and spun at 500 × g for 10 min. The SVF pellet was resuspended in DMEM/F12 medium (Lonza, Austria), filtered through a 100 μm and 40 μm nylon mesh cell strainer (VWR, Austria)^49^, counted with a CASY^TM^ cell counter (Schärfe System, Germany) and plated at a density of 10000 cells/cm^2^ for culture in PM4 medium^50^ containing DMEM/F12 (PAN Biotech, Germany) supplemented with 1 ng/ml rhFGF2, 10 ng/ml EGF (Immunotools, Germany), 50 ng/ml Insulin (Roche, Austria), 2.5% FCS and 1% Penicillin/Streptomycin (GE Healthcare, Austria). Twenty-four hours after plating non adherent cells were washed off and attached cells were assessed for multipotency. For FACS-sorting, SVF-cells were stained with CD31-FITC, CD45-FITC, CD34-PE (Biolegend, UK), CD90-APC (eBioscience, Austria) and 7AAD (BD Pharmingen, Germany) and sorted on a FACS Aria II cell sorter (Becton Dickinson, Germany). The CD34^+^/CD90^+^/CD31^−^/CD45^−^/7AAD^−^ ASC-subfraction^51^ was directly subjected to RNA- and protein-isolation.

### Isolation of lipid droplet containing adipocytes by isopycnic centrifugation

Lipid-droplet containing cells were separated from undifferentiated cells using a modified density centrifugation protocol with a 6% OptiPrep^®^ −0.5% FCS/PBS gradient. The gradient was generated by mixing 3 ml of OptiPrep^®^ (Sigma Aldrich, Germany) with 11 ml of a 0.5% FCS/PBS solution. After centrifugation (30 min, 800 × g) at 20 °C, trypsinized, *in-vitro* differentiated cells were layered on top of the gradient and spun for 30 min at 800 × g. The upper phase (~0.5 ml) containing adipocytes with lipid-droplets and the cell pellet (undifferentiated cells without lipid droplets) were directly lysed in laemmli buffer and subjected to immunoblotting. All reagents were obtained from Sigma Aldrich, Germany.

### Immunohistochemistry

Sections of paraformaldehyde fixed human tissue samples from fresh cadavers donated to the Department of Anatomy, Histology and Embryology of the Medical University of Innsbruck were stained with rabbit anti-ITGAV (ab179475, Abcam, UK), mouse anti-ITGA5 (MA5-15568, Thermo Scientific, Austria), mouse anti-Fibronectin (MS-1351, Thermo Scientific, Austria) or rabbit anti-PLIN1 ((D1D8) XP, cell signaling, USA) antibodies applying a Ventana Roche Discovery Immunostainer (Ventana, Germany) according to the DAB-MAP discovery research standard procedure. A biotinylated immunoglobulin cocktail of goat anti-mouse-IgG, goat anti-mouse-IgM, goat anti-rabbit-IgG and protein block (760–4205, Ventana) was applied for 30 min at room temperature. Hematoxylin (760–2021, Ventana) counterstained sections were manually dehydrated in downgraded alcohol series, cleared in xylene and permanently cover slipped with Entellan^®^ (Merck, Germany). Positive controls (placenta, liver) and negative control slides were added to each experiment to validate the IHC staining reactions. Digital images were acquired using AxioVision microscope software linked to an AxioCamHRc camera and an AxioPlan2 microscope (Zeiss, Germany). Because the dead bodies were immediately anonymized no certificate of non-objection was needed.

### Proliferation assay

Cell proliferation was determined by resazurin-based PrestoBlue assay (Invitrogen, Germany) by measuring mitochondrial activity^52^. Cells were seeded in 96 well plates (5000 cells per well). PrestoBlue reagent was added at a concentration of 0.1 mg/ml in PM4. Cells were incubated for 15 min at 37 °C. Fluorescence was measured at 530 nm on a Tecan Genios spectrophotometer (Tecan, Germany) according to the manufacturer’s instructions.

### Adipogenic differentiation

Adipogenic differentiation was induced using adipogenic induction medium (AIM) based on DMEM/F12 medium supplemented with 33 μM biotin, 1 μM troglitazone, 250 μM 3-isobutyl-1-methyl-xanthine (IBMX), 10 μg/ml transferrin, 200pM T3, 100 nM dexamethasone and 500 nM insulin. After 72 h, AIM was exchanged for adipocyte differentiation medium (ADM; AIM without IBMX) and cells were cultured until day 14. To induce chondrogenic differentiation, high density seeded cells were cultured for three weeks in StemPro basal medium containing StemPro chondrogenesis supplements (ThermoScientific, Austria). Osteogenic differentiation was induced by exposing proliferating cells (5000 cells/cm^2^) to osteogenic differentiation medium (DMEM, 10% FCS, 100 nM dexamethasone, 50 μM ascorbic acid, 10 mM glycerol-2-phosphate) for three weeks. Cells were stained with Oil red O (ORO, adipogenesis), Alcian blue (chondrogenesis) or Alizarin red (osteogenesis). All reagents and dyes were obtained from Sigma Aldrich, Germany. For the assessment of intracellular lipid content, cells were fixed in 3% PFA for 10 min, washed with PBS and incubated with HCS LipidTOX™ Green Neutral Lipid Stain (ThermoScientific, Austria) for 30 min. Digital images were acquired using an inverse Zeiss Axiovert 200 M microscope (Carl Zeiss, Austria) linked to an CoolSNAP FX CCD-camera and analyzed with ImageJ 1.46r Software (National Institutes of Health, USA) by counting differentiated cells per visual field.

### Plasmid construction and lentiviral transduction

Complementary shRNA oligonucleotides directed against ITGAV or ITGA5 (Supplementary table 1) were designed with Dharmacon siDesign-CENTER software, annealed and cloned into the BglII-HinDIII sites of pENTR-THT^27^. The sequence verified THT-shRNA cassette was recombined into the lentiviral RNAi destination vector pHR-Dest-SFFV-Puro as previously described^27^. For constitutive ITGAV or ITGA5 overexpression, ITGAV-cDNA isoform 2 (pEF1-human ITGAV-V5-His6^53^) was cloned into the BamHI/NotI site of pHR-SIN-CSGW-ΔNot^54^ replacing the eGFP cDNA. KpnI-digested and blunt ended ITGA5-cDNA from pEGFP-N3-ITGA5^55^ was cloned into the blunt ended BamHI/NotI site of pHR-SIN-CSGW-ΔNot. For survivin overexpression, survivin-cDNA (survivin variant 1 (NM_001168)) was cut from pLIB-survivin-IRES-YFP^56^ (provided by M. Ausserlechner) using EcoRI and NotI and cloned into the BamHI/NotI site of pHR-SIN-CSGW-ΔNot. EcoRI and BamHI sites were blunt ended using a DNA-polymerase-I Klenow-fragment. 1.5 μg of sequenced verified plasmids were then co-transfected with 0.9 μg pSPAX2 packaging and 0.9 μg pMD-G VSV-G pseudotyping plasmids using calcium phosphate transfection^57^. Twenty-four and 48 hours post transfection, sterile filtered supernatants were diluted 1:2 with fresh PM4 medium and supplemented with 1 μg/ml polybrene for infection; 48 hours after infection ASCs were selected for puromycin resistance (1 μg/ml) and analyses were performed five days after transduction. All reagents were obtained from Sigma Aldrich, Germany.

### RNA isolation and quantitative RT-PCR

RNA was isolated using TRIzol (MRC Inc. Cincinnati, OH, USA) and cDNA was synthesized using random hexamer primers and an iScript cDNA-synthesis kit (Biorad, Germany). The quantitative RT-PCR reactions were performed using the SsoAdvanced™ Universal SYBR^®^Green Supermix kit (Biorad, Germany) in a realplex-2 lightcycler (Eppendorf, Germany) using the following protocol: 95 °C for 3 min, 40 cycles at 95 °C (15 s), 60 °C (15 s), and 72 °C (10 s). Gene expression was estimated using the Mastercyler ep-realplex software version 2.2. CT values and normalized to the geometric mean of the reference genes glucuronidase beta (GUSB) and tyrosine 3-monooxygenase/tryptophan 5-monooxygenase activation protein zeta (YWHAZ)^58^. Primers (see Supplementary Table 1) were designed using NCBI Primer-blast software, and specificity was tested by the assessing the melting curve.

### Immunoblotting

For immunoblotting 2.5 × 10^5^ to 1 × 10^6^ cells were lysed in laemmli buffer containing 5% 2-β-mercaptoethanol (Sigma Aldrich, Germany), sonicated and boiled for 5 min at 95 °C. Proteins were size fractionated on prestained gradient polyacrylamide gels (Mini-PROTEAN^®^TGX Stain-Free™ Precast Gels, Biorad, Germany), blotted onto 0.2 μm PVDF membrane, blocked 2 h in 5% low fat milk powder and incubated overnight with primary antibodies against ITGAV (BD Biosciences, Germany), ITGA5 (Biolegend, USA), AKT, phAKT (S473), phERK (Thr202/Tyr204), ERK, PLIN1, FABP4, YAP, TAZ (all from Cell signaling, USA), p21^Cip1^ (ThermoScientific, Austria), survivin (Enzo Lifesciences, Switzerland), p73 (Imgenex, USA), p53 (MyBiosource, USA), GAPDH (6c5,Santa Cruz, Germany). After washing, horseradish peroxidase-conjugated sheep-anti-mouse and sheep-anti-rabbit antibodies (all from Cell signaling, USA) were incubated for 1 h and the reaction was visualized by enhanced chemiluminescence reagent ECL (Biorad, Germany) using a Biorad ChemidocMP gel analyzer for detection. Quantification was performed with the ImageLab 5.0 software (Biorad, Germany) according to the manufacturer’s instructions. Total protein loading was visualized using the ChemidocMP gel analyzer and employed as an internal loading control for all immunoblots^59^.

### Flow cytometry

Transduced ASCs were stained for integrin surface expression with ITGAV-PE and ITGA5-PE antibodies (Biolegend, UK) in PBS/2 mM ETDA/0.5% BSA according to the manufacturer’s instruction and analyzed on a Calibur FACScan cytometer using CellQuestPro software 4.0.1 (BD Biosciences, USA). Cell death was determined by analysis of AnnexinV-FITC/propidium iodide stained cells using an AnnexinV-FITC staining kit (BD Pharmingen, Germany) according to the manufacturer’s instructions. To determine adipogenic differentiation by flow cytometry, *in-vitro* differentiated adipocytes were stained using HCS LipidTOX™ Green Neutral Lipid Stain (Thermo Scientific, Austria) according to the manufacturer’s instructions. In short, cells were stained with LipidTOX™/PBS diluted 1:500 for 20 min, trypsinized and finally subjected to FACS analysis.

### Statistics

All experiments were repeated independently at least three times using cells from different donors. To assess quality of data, descriptive statistics were performed. Data were analyzed with unpaired Student’s t-test and the Mann-Whitney-U test and are presented as mean +/−SD or SEM. P-values <0.05 were considered statistically significant. Statistical analyses were performed using Statview software (SAS Institute Inc., version 5.0.1) and Prism 5 (Graphpad Software Inc., version 5.0).

## Additional Information

**How to cite this article**: Morandi, E. M. *et al.* ITGAV and ITGA5 diversely regulate proliferation and adipogenic differentiation of human adipose derived stem cells. *Sci. Rep.* **6**, 28889; doi: 10.1038/srep28889 (2016).

## Supplementary Material

Supplementary Information
