# Supplementary material for: ITGAV and ITGA5 diversely regulate proliferation and adipogenic differentiation of human adipose derived stem cells
Source: Sci Rep. 2016 Jul 1;6:28889. doi: 10.1038/srep28889 (PMC4929468; doi:10.1038/srep28889)
Supplement: Supplementary Information [file srep28889-s1.pdf]

Supplementary Information:

ITGAV and ITGA5 diversely regulate proliferation and adipogenic differentiation of human adipose derived stem cells

E. M. Morandi, R. Verstappen, M. E. Zwierzina, S. Geley, G. Pierer and C. Ploner*

Supplementary Figure 1: **Determination of ASC multipotency by trilineage differentiation.** Isolated ASC (A) subjected to adipogenic (B), chondrogenic (C) and osteogenic (D) differentiation were stained using ORO , Alcian blue and Alizarin red after 14 days (adipocytes) or 3 weeks of differentiation (chondrocytes and osteoblasts). Scales B, D: 25µm, C: 50µm. Quantitative RT-PCR of differentiation marker genes (ADIPOQ for adipocytes, SOX9 for chondrocytes and RUNX2 for osteoblasts) was carried out using cDNA isolated from differentiated cells (E). Expression data were normalized to the mean of the reference gene GUSB. Shown data represent the mean±SD of 3 donors. Asterisks indicate p-values <0.05.

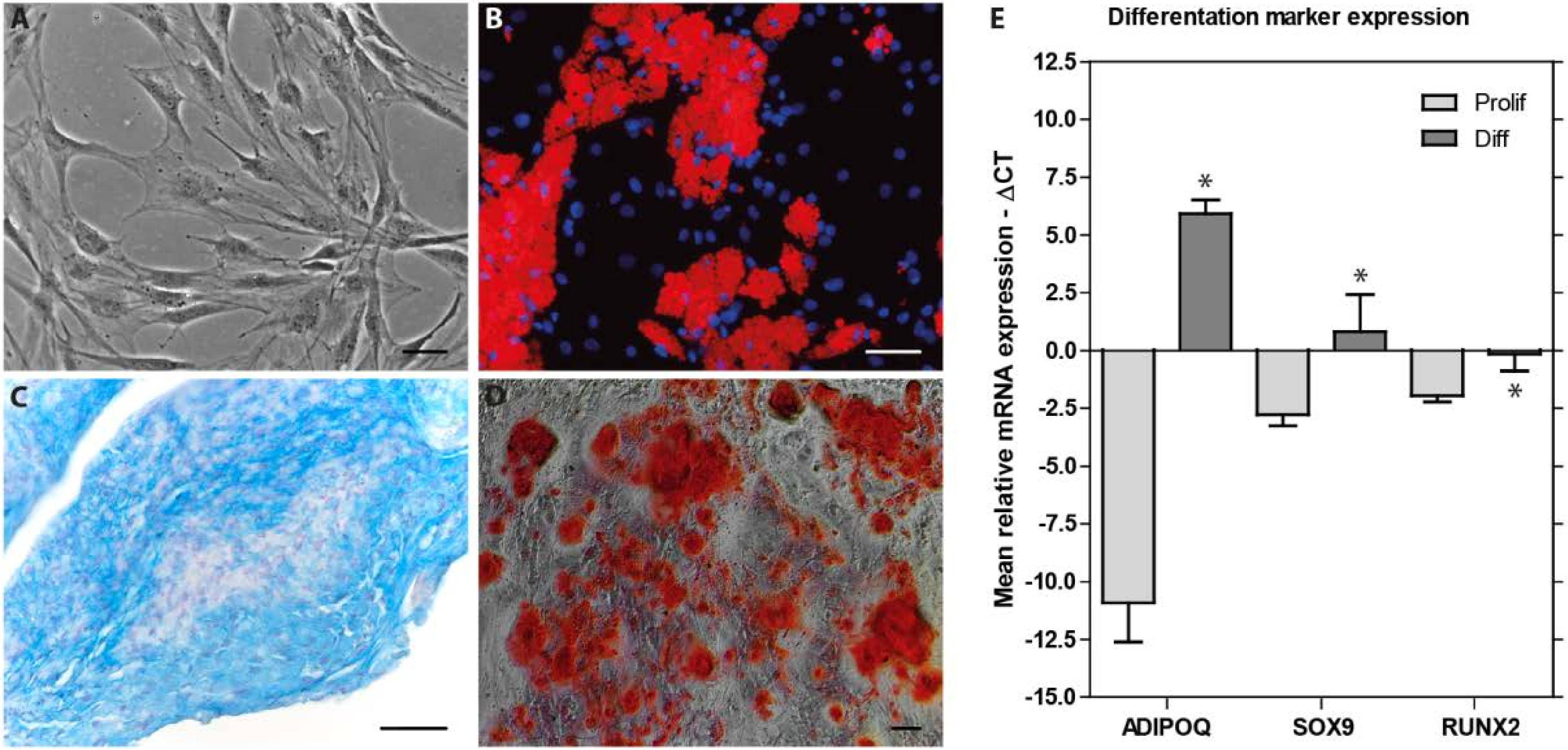

Supplementary Figure 2: **Effect of ITGAV- and ITGA5-KD on proliferation at a later time point.** Infected cells were cultured for 12 days after transduction and cell numbers were determined by automated cell counting (CASY^{TM}). Shown data represent the mean±SD of 3 experiments. Data of the KD groups have been pooled. Asterisks indicate p-values <0.05.

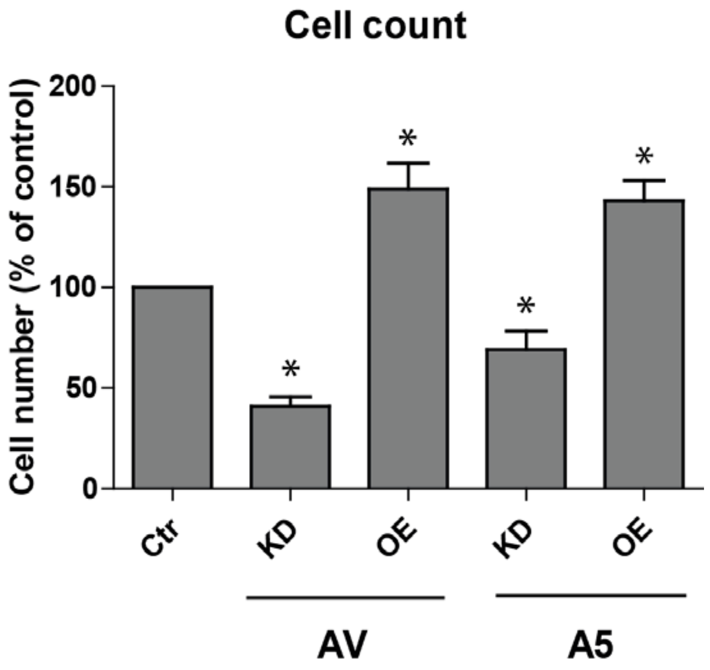

Supplementary Figure 3: **Identification of adipocytes in human fat tissue sections.** Immunohistochemistry analysis of paraffin fixed human subcutaneous fat tissue sections using specific antibodies detecting adipocyte marker PERILIPIN 1 (PLIN1) was used to mark mature adipocytes. Adipocytes can be identified morphologically as "signet ring cells" containing large fat vacuoles in the images and show a specific intracellular, peripheral staining of PLIN1. The images show protein expression visualized using horseradish peroxidase (brown) and hematoxylin counterstaining (blue), scale bars= 100µM, 10x magnification (A) and 25µM, 40x magnification (B).

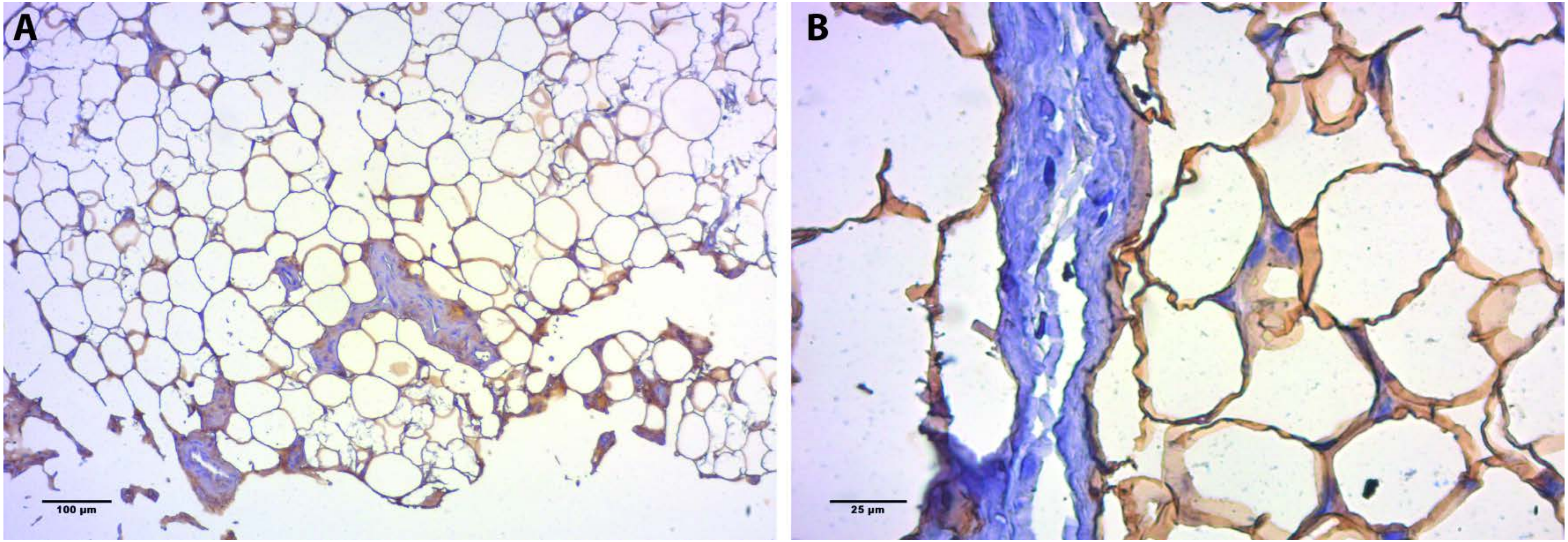

Supplementary Figure 4: **Representative protein loadings of depicted immunoblots – full length gels.** Proteins were size fractionated on prestained gradient polyacrylamide gels (Mini-PROTEAN®TGX Stain-Free™ Precast Gels, Biorad, Germany) and visualized using a ChemidocMP gel analyzer before blotting. Shown are full length gels of figure 2D, 2E, 3A and 4A as representative gels performed in this study. Of note, protein lysates from cells enriched by the Optiprep-method show a different protein pattern compared to classical harvested cells. However, this did not influence detectability of the reference gene GAPDH or the adipocyte marker genes FABP4 and PLIN1 (Fig. 2E).

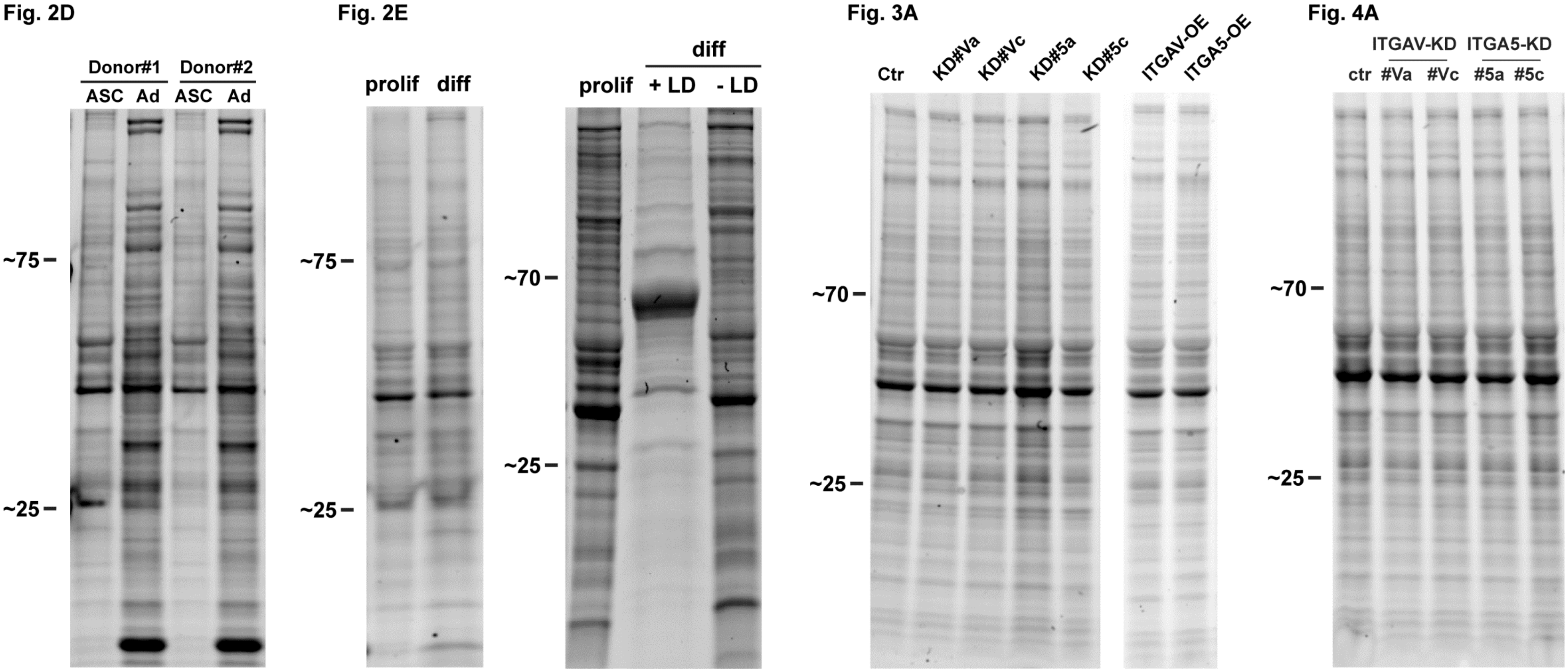

Supplementary Table 1: **Quantitative RT-PCR primer and RNAi:** The table lists all primers and oligonucleotides used in this study. Gene targeting sequences in shRNA oligonucleotides are underlined.

**Code**	**Sequence**
ITGA1 forward	5' - TGGTGGTGCTGCCCTCTTCTG - 3'
ITGA1 reverse	5' - GTGAATCTAGGGTGACACGGTACTGC - 3'
ITGA2 forward	5' - CCTTGAAGCCTATTCTGAGACTGCC - 3'
ITGA2 reverse	5' - AATTCCAGTGTTGTATGCACTTTCCC - 3'
ITGA3 forward	5' - ACTGTGAAGGCACGAGTGTGGAAC - 3'
ITGA3 reverse	5' - ATGCTGGTTCGGAGGAATAGGG - 3'
ITGA4 forward	5' - AGAGAGACAATCAGTGGTTGG - 3'
ITGA4 reverse	5' - TCAGTTCTGTTCGTAAATCAGG - 3'
ITGA5 forward	5' - TGCCTCCCTCACCATCTTC - 3'
ITGA5 reverse	5' - TGCTTCTGCCAGTCCAGC - 3'
ITGA6 forward	5' - GCTGGTTATAATCCTTCAATATCAATTGT - 3'
ITGA6 reverse	5' - TTGGGCTCAGAACCTTGGTTT - 3'
ITGA7 forward	5' - ATGGCTGTGGTGGCAGAAGGAG - 3'

ITGA7 reverse	5' - GAATCTTCACCGCATGGTACTGGG - 3'
ITGA8 forward	5' - CATCACTGCTGTAGCGCAGGTGG - 3'
ITGA8 reverse	5' - GTTCCACCAATGGTCCAACCTCC - 3'
ITGA9 forward	5' - CCATTGATGTGGTAGGAGGTGCC - 3'
ITGA9 reverse	5' - CACAAGGAGGAGCCGAAGTAAGAGC - 3'
ITGA10 forward	5' - CATCCTGTCTTCCAGACTGGAGCC - 3'
ITGA10 reverse	5' - TGGATGTAGGCTGAGGTCTGGGC - 3'
ITGA11 forward	5' - ACCAATGGCTACCAGAAGACGGG - 3'
ITGA11 reverse	5' - GGCCGAGGCGCATGTTGTC - 3'
ITGAD forward	5' - GATGAGGGCCTAAGAAGCAGCCG - 3'
ITGAD reverse	5' - GGTGGCCTTGCTGCTTGAAGC - 3'
ITGAE forward	5' - TGAGGGACTGAATGCAGAGAAC - 3'
ITGAE reverse	5' - CAACACCAGAAGTCCACCAAC - 3'
ITGAL forward	5' - GGGGGGACTCGGTTGAATTGC - 3'
ITGAL reverse	5' - GTGCTTGACTTGGTGGATCTTGGG - 3'
ITGAM forward	5' - GTGAAGCCAATAACGCAGCTGC - 3'
ITGAM reverse	5' - TGTCTGCCTCAGGGATGACATCC - 3'

ITGAV forward	5' - GGATTGTTGCTACTGGCTGTTTTGG - 3'
ITGAV reverse	5' - TCCCTTTCTTGTTCTTCTTGAGGTGG - 3'
ITGAX forward	5' - CCTGTCCCTGGCGTCTACCACC - 3'
ITGAX reverse	5' - ATGTCCTGCTCCTGTCTTGGGC - 3'
YWHAZ forward	5' - ACTTGACATTGTGGACATCGGA - 3'
YWHAZ reverse	5' - GTGGGACAGCATGGATGACA - 3'
GUSB forward	5' - GGAATTTTGCCGATTTCATGAC - 3'
GUSB reverse	5' - TCTCTGCCGAGTGAAGATCCC - 3'
TBP forward	5' - GCCACGAACCACGGCACTG - 3'
TBP reverse	5' - TTTCTTGCTGCCAGTCTGGACTG - 3'
FABP4 forward	5' - TCAGTGTGAATGGGGATGTGATC - 3'
FABP4 reverse	5' - TCAACGTCCCTTGGCTTATGC - 3'
PPARG forward	5' - CACAAGAACAGATCCAGTGGTTGCAG - 3'
PPARG reverse	5' - AATAATAAGGTGGAGATGCAGGCTCC - 3'
ADIPOQ forward	5' - GATGGCAGAGATGGCACCC - 3'
ADIPOQ reverse	5' - GGAATTTACCAGTGGAGCCA - 3'
PLIN2 forward	5‘- CCTGTAAGGGGCTAGACAGGA - 3‘

PLIN2 reverse	5' - GTCACAGTAGTCGTCACAGCA - 3‘
SURVIVIN1 forward	5' - TTCAAGGAGCTGGAAGGCTG - 3'
SURIVIN1 reverse	5' - GCAACCGGACGAATGCTTTT - 3'
P21 forward	5' - GTACCCTTGTGCCTCGCTC - 3'
P21 reverse	5' - GCGGATTAGGGCTTCCTCTTG - 3'
CTGF forward	5' - GGAGTGGGTGTGTGACGAGCC - 3'
CTGF reverse	5' - GGACCAGGCAGTTGGCTCTAATC - 3'
RUNX2 forward	5' - CCAAATTTGCCTAACCAGAA - 3'
RUNX2 reverse	5' - GCTCGATTGCAATTGTCTCT - 3'
SOX9 forward	5' - GTACCCGCACTTGCACAAC - 3'
SOX9 reverse	5' - TCGCTCTCGTTCAGAAGTCTC - 3'
ITGAV-shRNA#a	5’-ATCCCCGGTCAAGATCAGTGAGAAATTCAAGAGATTTCTCACTGATCTTGACCTTTTTGGAAA - 3'
ITGAV-shRNA#c	5' – GATCCCCGGTGAACCTTCTAGAGGAATTCAAGAGATTCCTCTAGAAGGTTCACCTTTTTGGAAA - 3'
ITGA5-shRNA#a	5' - ATCCCCTGGACAAGGCTGTGGTATATTAAGAGATATACCACAGCCTTGTCCATTTTTGGAAA - 3'
ITGA5-shRNA#c	5' – GATCCCCGAGAGGAGCCTGTGGAGTATTCAAGAGATACTCCACAGGCTCCTCTCTTTTTGGAAA - 3'
Ctr-shRNA	5' – GATCCCCAATAGCGACTAAACACATCAATTCAAGAGATTGATGTGTTTAGTCGCTATTTTTTTGGAAA - 3'

Supplementary Methods:

Protein isolation from in-vivo differentiated adipocytes

Proteins from primary adipocytes were isolated by resuspending approximately $2x10^6$ cells in 500µl isolation buffer containing 50mM Tris, 150mM NaCl, 0.2mM ETDA and supplemented with protease inhibitor (Roche, Germany) and 1875µl chloroform/methanol (1:2 mix). After homogenization and incubation on ice for 10min, 625µl chloroform and 625µl aqua bidest were added. The lysates were spun at 800xg for 5 minutes and the protein disks (middle layer) was resupended in 0.1% SDS, 40mM Tris, mixed with appropriate amount of laemmli buffer supplemented with 5% beta-mercaptoethanol and boiled for 5 minutes.
